# Cannabinoid receptor 2 evolutionary gene loss makes parrots more susceptible to neuroinflammation

**DOI:** 10.1098/rspb.2022.1941

**Published:** 2022-12-07

**Authors:** Daniel Divín, Mercedes Goméz Samblas, Nithya Kuttiyarthu Veetil, Eleni Voukali, Zuzana Świderská, Tereza Krajzingrová, Martin Těšický, Vladimír Beneš, Daniel Elleder, Oldřich Bartoš, Michal Vinkler

**Affiliations:** ^1^ Faculty of Science, Department of Zoology, Charles University, Viničná 7, Prague 128 44, Czech Republic; ^2^ Genomics Core Facility, European Molecular Biology Laboratory Heidelberg, Meyerhofstrasse 1, 69117 Heidelberg, Germany; ^3^ Institute of Molecular Genetics, Czech Academy of Sciences, Vídeňská 1083, 142 20 Prague 4, Czech Republic; ^4^ Military Health Institute, Military Medical Agency, Tychonova 1, 160 01 Prague 6, Czech Republic

**Keywords:** neuroimmunology, avian immunology, cannabinoid receptors, neural inflammation, gene loss, cannabinoid receptor pseudogenization

## Abstract

In vertebrates, cannabinoids modulate neuroimmune interactions through two cannabinoid receptors (CNRs) conservatively expressed in the brain (CNR1, syn. CB1) and in the periphery (CNR2, syn. CB2). Our comparative genomic analysis indicates several evolutionary losses in the *CNR2* gene that is involved in immune regulation. Notably, we show that the *CNR2* gene pseudogenized in all parrots (Psittaciformes). This *CNR2* gene loss occurred because of chromosomal rearrangements. Our positive selection analysis suggests the absence of any specific molecular adaptations in parrot *CNR1* that would compensate for the *CNR2* loss in the modulation of the neuroimmune interactions. Using transcriptomic data from the brains of birds with experimentally induced sterile inflammation we highlight possible functional effects of such a *CNR2* gene loss. We compare the expression patterns of CNR and neuroinflammatory markers in *CNR2*-deficient parrots (represented by the budgerigar, *Melopsittacus undulatus* and five other parrot species) with *CNR2*-intact passerines (represented by the zebra finch, *Taeniopygia guttata*). Unlike in passerines, stimulation with lipopolysaccharide resulted in neuroinflammation in the parrots linked with a significant upregulation of expression in proinflammatory cytokines (including interleukin 1 beta (*IL1B*) and 6 (*IL6*)) in the brain. Our results indicate the functional importance of the *CNR2* gene loss for increased sensitivity to brain inflammation.

## 1. Introduction

Psychiatric and neurodegenerative disorders have increased in incidence globally in the human population [1,2]. Surprisingly, similar psychological (in animals referred to as behavioural) disorders have been frequently recognized in some cognitively advanced animals, namely the parrots [3–5]. Like in human depression, in parrots symptoms like anxiety, apathy, over-eating, indifference and self-damage (feather plucking) are observed and diagnosed by veterinarians [6–8]. While in parrots little is presently known about the possible causes of the behavioural disorders, in humans they have been recently linked with neural inflammation [9]. Brain neuroinflammation can be induced by signals from the periphery, where pathogens and tissue damage trigger immune responses through stimulation of pattern recognition receptors [10]. Resultant cytokine signalling may modulate central nervous system functioning through the activation of brain microglia and astrocytes [11] interfering with healthy brain neuronal regulation [12,13]. Proinflammatory cytokines, such as interleukin 1 beta (IL1B) or 6 (IL6) [14,15] become overexpressed in the brain, being key neuroinflammation markers [16,17]. Mental health depends on finely balanced regulation of the neuroimmune interplay. Among neuronal modulators interlinking the nervous and immune systems, cannabinoids recognized through cannabinoid receptors (CNRs) have been shown to provide important anti-neuroinflammatory effects in humans [18–21]. Compared to humans, in animals the immunological factors affecting behavioural disorders are far less clear and interspecific variation in the neuroimmune regulatory networks remains unknown. Parrots with their advanced cognitive abilities [22], dense neuronal networks [23] and common psychopathologies [6,7] could help us to understand the general principles of neuroinflammation effects on behaviour.

Gene loss is a widespread phenomenon responsible for evolutionary changes in organisms, including immunity and other physiological functions [24]. It may be involved in adaptive responses to environmental or pathogen-driven changes in selective pressures [25] or represent a random shift in gene content with deleterious effects insufficient to be prevented by negative selection [26]. Genomic chromosomal rearrangement is probably an important source of the gene loss events. Massive chromosomal alterations have profoundly affected vertebrate evolution in general [27], as well as in certain lineages [28] including parrots in particular [29–31]. Recent advances in genomic research have allowed thorough mapping of evolutionary gene loss events affecting immune signalling [24,32–35]. In the present study, we performed a comparative genomic database search of negative regulators of neural inflammation which indicated an interesting pattern of loss in the *CNR2* gene in parrots.

The endocannabinoid system regulating both the neural and immune functions consists of CNRs, their ligands (endocannabinoids) and enzymes synthesizing and degrading cannabinoids [36]. Two CNR paralogues are known in all vertebrates [37]: *CNR1*, which is mainly expressed in cells of the nervous system, and *CNR2*, which is mainly expressed in immune cells, including microglia in the brain [38,39]. CNR1 is involved in the regulation of emotions, memory, motor activity, feelings, attention, neuropeptide synthesis, gastrointestinal tract functions, metabolism and—in birds—singing [40–44]. CNR2 affects immunosuppression and decreases inflammation, pain and the expression of proinflammatory cytokines, playing an important role in negative feedback regulation [45–47]. *CNR2* expression has been shown to increase with the activation of immune cells related to higher expression of proinflammatory cytokines [39]. In mice, *CNR2* expression in brain-based microglia was upregulated during neurological inflammation, contributing to the suppression of the inflammatory response [45].

Since our comparative genomic search suggested that among negative regulators of inflammation, parrots consistently miss only the *CNR2* gene, here we use genomic and transcriptomic data to map the putative *CNR2* loss events across vertebrate phylogeny. Subsequently, exemplified in the budgerigar (*Melopsittacus undulatus*) and kakapo (*Strigops habroptila*) genomes we reconstruct the *CNR2* loss events in parrots. Using analysis of positive selection, we search for compensatory adaptations in *CNR1* in species lacking CNR2. Finally, by comparing parrot and passerine neuroinflammation marker expression patterns in the brain during an immune response, we assess the consequences of *CNR2* loss on neuroimmune regulation in parrots.

## 2. Methods

### (a) Identification of cannabinoid receptor-loss events

To identify the candidate genomic differences between passerines and parrots that could result in parrot susceptibility to neuroinflammatory disorders, we first checked for the patterns of gene representation in these two taxa. The gene set of negative regulators of inflammatory responses (GO:0050728) was retrieved from the AmiGO database, accessed August 2022 [48], based on human (*Homo sapiens*) annotation (*n* = 154) and then chicken (*Gallus gallus*) orthologues, identified using the ENSEMBL BioMart tool [49]. This allowed us to shortlist 122 avian genes involved in the regulation. Using the Avian Immunome database (AVIMM; accessed August 2022 [50]) we identified 53 immune genes to which information on presence/absence could be retrieved across six parrot and 13 passerine species covering the Psittacopasserae phylogeny: *Melopsittacus undulatus*, *Eolophus roseicapillus*, *Probosciger aterrimus*, *Amazona guildingii*, *Agapornis roseicollis*, *Nestor notabilis*, *Corvus moneduloides*, *Ficedula albicollis*, *Hirundo rustica*, *Lepidothrix coronata*, *Lonchura striata*, *Molothrus ater*, *Parus major*, *Passer domesticus*, *Serinus canaria*, *Sturnus vulgaris*, *Taeniopygia guttata*, *Zonotrichia albicollis*, *Zosterops hypoxanthus*. In this list, we checked for cases of consistent absence of a regulating gene in parrots and its consistent presence in passerines. Only a single gene, *CNR2*, fulfilled this criterion (electronic supplementary material, S2, table S21).

For the phylogenetic analysis of the two related CNR genes, *CNR1* and *CNR2*, we first downloaded all available tetrapod *CNR* coding DNA sequences from the Ensembl genome browser database (release 103, www.ensembl.org; last accessed on 22 January 2021). Based on a comparison of lists of species with annotated *CNR1* and *CNR2*, we identified all cases of putative *CNR1* or *CNR2* absence. For these species, we performed a targeted search through the NCBI databases (https://www.ncbi.nlm.nih.gov, release 236) using blastx and tblastn (https://blast.ncbi.nlm.nih.gov/Blast.cgi) to find the missing orthologues. Using this complete sequence dataset, supplemented with *CNR1* sequences from five other parrots species represented in the parrots′ experiment (E2) obtained by Next Seq Illumina transcriptomic sequencing (see below), we reconstructed the *CNR* phylogenetic tree (based on 318 sequences) in the online tool iTOL to verify the sequence gene-specific orthology [51,52]. For a list of all species, including their *CNR1* and *CNR2* sequence accession numbers, see the electronic supplementary material, S1, table S1. The final dataset consisted of 160 orthologues of zebra finch (*T. guttata*)/budgerigar *CNR1* and 158 orthologues of zebra finch *CNR2* (electronic supplementary material, table S1). The position of *CNR2* in the zebra finch, chicken and human karyotypes was checked in Ensembl and the neighbouring coding genes were identified in parrots with karyotype information available in Ensembl (the budgerigar and kakapo). Based on this data, we reconstructed the genomic changes leading to *CNR2* pseudogenization.

### (b) Selection analysis

We examined the evidence for positive selection acting on vertebrate CNRs in order to infer whether loss of *CNR2* could be linked to any alteration to *CNR2* functioning in the clade of parrot-related taxa, and whether it might have resulted in any compensatory evolution in parrot *CNR1*. First, we used the tool CONSURF (http://consurf.tau.ac.il; [53] to identify non-conservative regions on the CNR surface. Next, we adopted a combination of tools for detecting positive selection available on the Datamonkey server (https://www.datamonkey.org/): FUBAR [54], MEME [55], aBSREL [56] and RELAX [57]; for details see the electronic supplementary material). We then used the online tools PROVEAN (Protein Variation Effect Analyzer, http://provean.jcvi.org; [58]) and SIFT (Scale Invariant Feature Transform, https://sift.bii.a-star.edu.sg/; [59]) to predict functional effects of the amino acid substitutions observed at sites under positive selection.

### (c) Experimental procedures

In budgerigars (experiment 1 (E1), *n* = 30), in the six selected parrot species (experiment 2 (E2), *n* = 36, i.e. the red-rumped parrot *Psephotus haematonotus*, the rosy-faced lovebird *Ag. roseicollis*, the elegant parrot *Neophema elegans*, the budgerigar, the cockatiel *Nymphicus hollandicus* and the pacific parrotlet *Forpus coelestis*, six individuals per species) and in the zebra finches (experiment 3 (E3), *n* = 24) we used standardized methodology to map the *CNR* and *IL1B* expression trajectories during acute immune response (see the electronic supplementary material, S1, table S2). All birds from all experiments (E1–3) were obtained from local hobby breeders and housed in pairs in cages 100 × 50 × 50 cm. The birds had access to food and water ad libitum and were kept under a 12 L : 12 D controlled light/dark cycle with a regulated temperature of 22 ± 2°C. Treatment individuals were intra-abdominally injected with lipopolysaccharide (LPS; *Escherichia coli O55:B5*; Sigma-Aldrich, cat. no. L2880) in a dose equivalent to 6 µg per gram body weight and compared to controls injected with a sterile Dulbecco's phosphate-buffered saline (Sigma-Aldrich, cat. no. D5652). The LPS dose was chosen based on previous studies in other small-sized birds inducing a measurable non-specific immune response [60]. In the first experiment with budgerigars (E1) the experimental birds were euthanized at different time points, i.e. at 3, 6, 12, 24 and 48 h post-treatment (*n* = 3 per time point and treatment) to check for the immune response dynamics, while in the second experiment with the different parrot species (E2) and in the third experiment with the zebra finches (E3) (consistent with the results from E1) the time interval for the immune response was set to 24 h. In all birds, tissue samples of the small intestine (ileum) and the brain hyperpallial area were collected as necropsies after euthanasia, placed immediately into RNA later (cat. no. 76106, Qiagen, Hilden, Germany) and stored at −80°C until RNA extraction. The research was approved by the Ethical Committee of Charles University, Faculty of Science (permits 13882/2011-30 and MSMT-30397/2019-5) and was carried out in accordance with the current laws of the Czech Republic and the European Union.

### (d) Transcriptomic search for *CNR1* and *CNR2* genes in parrots

Small intestine transcriptomes for the six parrot species were obtained from sequencing libraries prepared using the NEBNext Ultra II Directional RNA Library Prep Kit for Illumina (cat. no. E7760, San Diego, CA, USA) in the European Molecular Biology Laboratory (EMBL), Heidelberg (NCBI accession numbers: SAMN23963146, SAMN23963147, SAMN23963148, SAMN23963149, SAMN23963150, SAMN23963151). Paired-end sequencing (80 bp from each end) was performed on the NextSeq 500 system (Illumina) at a sequencing depth of 13–19 million reads per library. Forward and reverse reads were merged, and low-quality reads and adaptor sequences discarded, using BBsuite (‘BBMap’ n.d.). *De novo* transcriptome assembly was performed by Trinity [61] under default settings. To obtain sets of non-redundant transcripts, we applied two filtering steps. First, we used TransDecoder [62] to identify the longest open reading frame of each transcript for each species individually, and second, redundancy was further reduced in the remaining transcript sets by clustering highly similar sequences with CD-Hit [63], using a sequence identity threshold of 0.9. Completeness of the six assembled transcript sets against a set of highly conserved single-copy orthologues was assessed using BUSCO (Benchmarking Universal Single-Copy Orthologs v.4.1.4; [64]. To identify *CRN1* and *CRN2* coding sequences for each species, reference budgerigar (for *CRN1*, Ensembl transcript ID: ENSMUNT00000010298.1) and zebra finch (for *CRN2*, Ensembl transcript ID: ENSTGUG00000001188) sequences were searched using Blastn [65] and compared against raw reads and the sequences obtained for positive selection analysis, and further against transcriptome assemblies.

### (e) Brain transcriptomic gene expression analysis

As an initial check for the differential gene expression in selected cytokines in zebra finch and budgerigar brains, we used the QuantSeq 3′end sequencing approach [66]. Samples were first barcoded with Illumina TruSeq adapters and sequencing was undertaken on the Illumina Hiseq 2500 platform at EMBL, Heidelberg. The sequenced samples (NCBI accession number: PRJNA751848 and PRJNA879979) were then analysed using the BAQCOM pipeline (https://github.com/hanielcedraz/BAQCOM), the adapters being removed using the Trimmomatic tool (http://www.usadellab.org/cms/). The samples were then aligned to the zebra finch reference genome (downloaded from Ensemble) using STAR aligner (https://github.com/alexdobin/STAR), the featureCounts, Subread R package being used to assign read counts to the genes. Given their low representation in the transcriptomic data, specific inflammatory markers, *IL1B*, *IL6*, *IL8*, *IL12B, IL15, IL17B*, *IL18* and *IL22* were selected based on the literature review [67] and their 3′ annotation available in the Ensemble. In *IL6*, *IL17B* and *IL22* we did not obtain sufficient read coverage to proceed further with a quantitative analysis. In order to normalize the expression data in the rest of the target genes, we first divided the total number of reference (cytokine)-aligned reads by the total number of reads in the sample (Cn). To scale the data, we then multiplied each of the normalized read counts by 10 million (approx. 10 million was the average number of reads per sample in our dataset). The cytokine expression was quantified as the scaled-normalized number of reads per treatment individual divided by the mean scaled-normalized number of reads in all the control birds: relative differential gene expression = (Cn × 10^6^)_Treatment_/(Σ(Cn × 10^6^)/*N*)_Control_.

### (f) Real-time quantitative polymerase chain reaction gene expression analysis

Designing conserved primers based on avian interspecific alignments we amplified the partial coding regions of *IL1B*, *IL6*, *CNR1* and *CNR2* and Sanger sequenced these in genomic DNA (gDNA) extracted from 12 blood samples representing different parrot species, 10 budgerigar samples and 12 zebra finch samples to assess intraspecific genetic variability and to design conserved real-time quantitative polymerase chain reaction (RT-qPCR) primers (electronic supplementary material, S1, table S3). The sequences were analysed using Geneious (http://www.geneious.com, [68]).

Total RNA was extracted from parrot and zebra finch brain samples using the High Pure RNA Tissue Kit (cat. no. 12033674001; Roche, Basel, Switzerland), the concentration and quality of the RNA being measured on the NanoDrop 1000 Spectrophotometer (Thermo Fisher Scientific). The RNA was diluted in molecular water enriched with carrier transfer RNA (Qiagen, cat. no. 1068337) in the ratio 1 : 5 for target genes or 1 : 500 for *28S rRNA*. To calculate the efficiency of each primer pair, a calibration curve was constructed with synthetic DNA standard (gBlocks; IDT, Coralville, IA, USA; electronic supplementary material, S1, table S4) using a dilution series of 10^8^–10^2^ copies µl^−1^, estimated according to Vinkler *et al.* [69]. The RNA samples and standards were amplified using the Luna Universal Probe One-Step RT-PCR Kit (E3006, BioLabs Inc, Ipswich, MA, USA), with 0.6 mM primer and 0.2 mM probe concentrations (electronic supplementary material, S1, table S5). RT-qPCR quantification was conducted using a LightCycler 480 PCR platform (Roche) set with the cycling conditions shown in the electronic supplementary material, S1, table S6. All assays were performed with template-free negative controls and block positive controls in a freshly prepared dilution series, using *28S rRNA* as a reference gene. Relative quantification (R) was calculated from the crossing point (Cp) values determined by the second derivative maximum [70], using E and Cp data calculated using LightCycler480 software v.1.5.1. To test for gene expression changes between treatment and control birds, we quantified relative gene expression as standardized relative quantities (Qst; [69]. For the RT-qPCR efficiencies (E) see the electronic supplementary material, S1, table S7; for the final RT-qPCR data see the electronic supplementary material, S2, table S22).

### (g) Statistical analysis

The statistical analysis was performed in Rstudio v.2021.09.0 [71]. First, the initial transcriptomic cytokine expression data were tested for the differences in inflammatory genes expression between the budgerigars and zebra finches using a Wilcoxon paired test and the results were plotted in a heatmap generated using the pheatmap package. Next, the RT-qPCR verification of these results was performed. Given their non-Gaussian distribution, the Qst values were normalized using decadic logarithms (logQst). The effects of experimental treatment on gene expression changes were assessed using the linear models (LMs) in the ‘Ime4’ package, where gene expression (continuous) served as a response variable. For the budgerigar (E1) dataset, the full model contained treatment, sex and time as explanatory variables. For the comparative parrot (E2) dataset, the full model contained treatment, sex and species as explanatory variables. Based on the E1 results, for the zebra finch (E3) dataset, only treatment was used as an explanatory variable in the full model. Minimum adequate models (here defined as models with all terms significant at *p* ≤ 0.05) were selected by backward elimination of non-significant terms from the full models. All backward elimination steps in the models were verified by changes in deviance with an accompanying change in degrees of freedom (ANOVA) and Akaike information criterion, using *F*-statistics. The Pearson correlation test was used to assess the relationship between the expression of the *CNR* genes and *IL1B* and *IL6*.

## 3. Results

### (a) Identification of the cannabinoid receptor genes in parrot genomes

**Figure 1.**
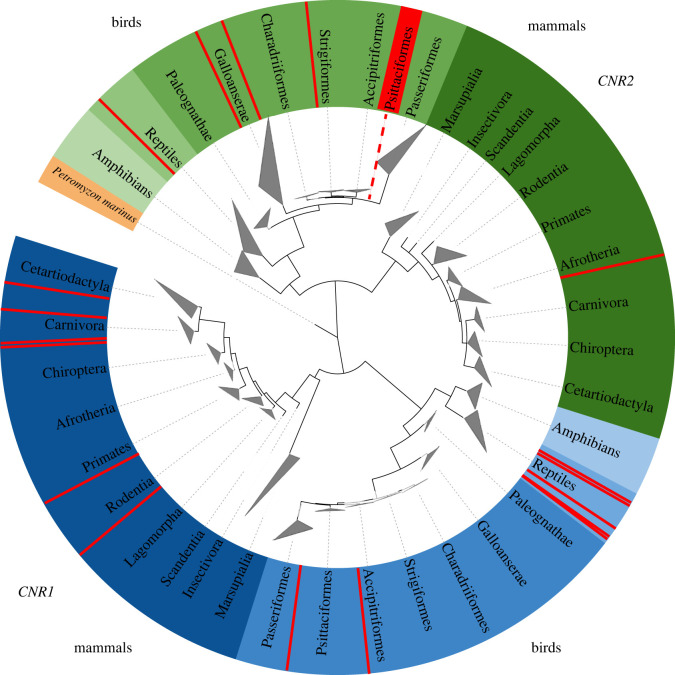
Phylogenetic tree showing gene-specific clustering of *CNR1* (blue) and *CNR2* (green). Lamprey (orange) shows the root of the tree as a common ancestor of the genes. Terminal triangles represent collapsed taxon-specific branches. The red colour highlights the presence of species with missing receptors (i.e. cases where the receptors were not revealed in the database search). A fully expanded tree is provided in the electronic supplementary material, figure S1).

**Figure 2.**
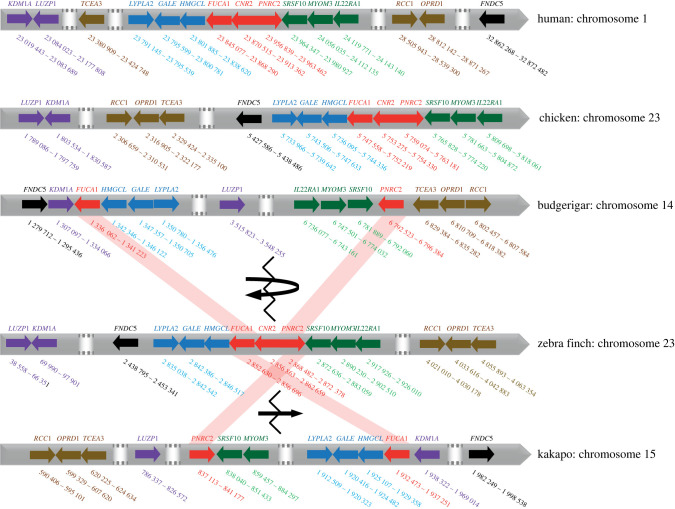
Schematic of the *CNR2* locus position and its neighbourhood in the human, chicken, budgerigar, zebra finch and kakapo genomes. Chromosome sequence is schematically indicated with the grey arrowed line over which positions of individual genes are indicated by colourful arrows (each colour represents one group of genes sitting in proximity in the ancestral state); *CNR2* and its closest human, chicken and zebra finch neighbouring genes, *FUCA1* and *PNRC2*, are marked in red and linked by red shades between the species. Each gene is labelled with its symbol above the arrow and its chromosomal location is marked below. White areas with the dotted grey lines indicate longer interspacing regions containing additional genes that are not important for the reconstruction of the chromosomal rearrangements in parrots. The recombination breakpoint in *CNR2* is indicated by a broken line, a curved arrow indicates the inversion event that occurred in the budgerigar evolutionary lineage, while a straight arrow indicates the translocation event that putatively occurred independently in the kakapo evolutionary lineage.

Searching through genomic databases, we identified a single negative regulator of neuroinflammation consistently missing in parrots, but consistently present in passerines, the *CNR2*. To confirm this pattern, we used the tetrapod *CNR* sequence data retrieved from Ensembl supplemented with the NCBI BLAST-search results (electronic supplementary material, S1, table S1) to construct a *CNR* phylogenetic tree showing *CNR1* and *CNR2* presence and absence (figure 1). We failed to identify the *CNR2* gene in any parrot species, though it was present in all parrot relatives: falcons (Falconiformes), seriemas (Cariamiformes) and passerines (Passeriformes). According to Ensembl, the *CNR2* gene is located on the 23rd chromosome in the zebra finch and chicken genomes, being directly adjacent to the *FUCA1* gene (upstream) and the *PNRC2* gene (downstream; figure 2). In the budgerigar genome, we found both these genes on chromosome 14; however, there was a approximately 5.5 Mbp insertion with inverted gene order directly between *FUCA1* and *PNRC2* (figure 2). Using BLAST, we identified short gene fragment showing 28% similarity to the barn owl (*Tyto alba*) *CNR2* and 10% similarity to the blue-crowned manakin (*Lepidothrix coronata*) *CNR2,* 6072 bp downstream of *PNRC2*. Interestingly, in the kakapo genome, different genes were situated downstream of the *PNRC2* gene (on the 15th chromosome; figure 2) and there was no sign of any remaining *CNR2* gene or pseudogene. To confirm the absence of the *CNR2* gene in parrot genomes, we designed sequence-conserved *CNR1* and *CNR2*-specific primers and sequenced the parrot gDNA-derived PCR amplicons. By contrast to the zebra finch, we found no evidence for the *CNR2* presence in budgerigar or any other parrot gDNA. Finally, our whole transcriptome complementary DNA sequencing in inflamed small intestine tissue failed to reveal *CNR2* in budgerigars, or in any of the other five parrot species analysed. We take this as conclusive evidence for the absence of functional *CNR2* in parrots.

### (b) Positive selection in cannabinoid receptors

We next questioned the hypotheses that the loss of *CNR2* could be linked to its altered function in the parrot-related taxa and that *CNR1* took over the functional role of *CNR2* when lost in the parrots. Across tetrapods, the test for selection relaxation was not significant in *CNR1* (*K* = 0.66, *p* = 0.822, LR = 0.05) or *CNR2* (*K* = 1.03, *p* = 0.964, LR < 0.001). Using CONSURF, we identified 67 non-conservative sites in *CNR1* and 61 non-conservative sites in *CNR2* (electronic supplementary material, figure S2; S1, tables S8 and S9). In *CNR1*, the FUBAR test failed to identify any positively selected sites, while the MEME test identified seven sites under episodic positive selection (electronic supplementary material, S1, table S10). In *CNR2*, one positively selected site was identified by FUBAR and 15 sites were revealed as under branch-specific positive selection by MEME (electronic supplementary material, table S10). However, no specific non-synonymous substitutions with a putatively compensatory role were identified in *CNR1* in parrots and there was no indication of any changes in *CNR2* function in parrot relatives. Also, aBSREL found no evidence of any episodic diversifying selection in parrot phylogeny in the *CNR1* gene or in parrot-related species (i.e. zebra finch, common kestrel) in the *CNR2* gene. PROVEAN used to identify significant changes in function caused by any amino acid variation, failed to indicate any important changes. Finally, SIFT predicted functional changes in *CNR1* at the sites D466R (with a score of 0.04) and T468I (score 0.04), and in *CNR2* at site V342I (score 0.05), but none of these changes proved important in birds. As such, we consider both CNR1 and CNR2 to be functionally conserved in the taxa where these genes are present.

### (c) Transcriptomic evidence for *CNR2*-associated variation in inflammatory marker expression changes in brain during an immune response

**Figure 3.**
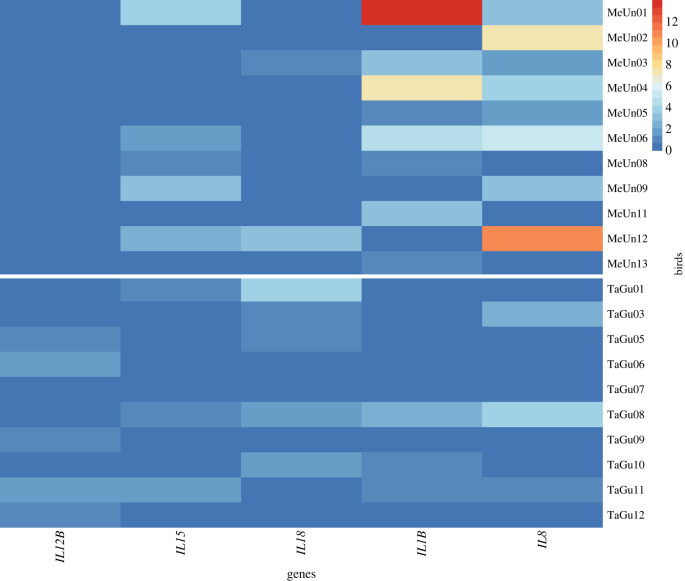
Heatmap showing differences in relative expression changes of proinflammatory cytokines in brains of LPS-stimulated budgerigars and zebra finches. MeUnX = budgerigar, X = number of individual; TaGuY = zebra finch, Y = number of individual. The heatmap colour ranges from blue (lowest upregulation in expression) to red (highest upregulation in expression).

We used transcriptomic data from zebra finch and budgerigar brains to check for the *CNR2*-linked functional variation in neuroinflammatory responsiveness. Checking for expression changes in the expression of proinflammatory cytokines after *in vivo* stimulation with LPS, we detected statistically significant differences between these two species in *IL1B* (Wilcoxon paired test; *p* = 0.021), *IL8* (*p* = 0.037), *IL12B* (*p* = 0.001) and *IL18* (*p* = 0.020) responses. For *IL6*, *IL17B* and *IL22* there was insufficient read representation to perform the statistical test and for *IL15* we found no significant difference in gene expression between the two species (*p* = 0.717). The results are shown in figure 3.

**Figure 4.**
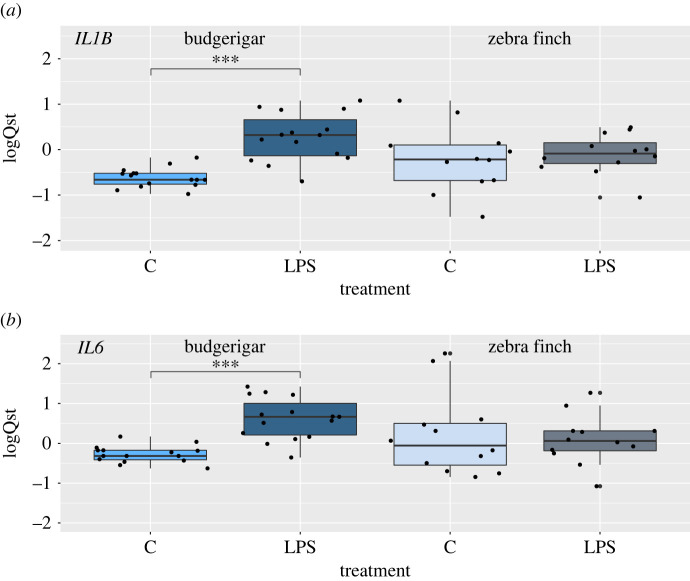
Expression of (*a*) *IL1B* and (*b*) *IL6* following peripheral stimulation with LPS in the brains of budgerigars and zebra finches. Gene expression assessed based on RT-qPCR is shown as centred standardized relative expression (logQst) values, i.e. species population average is equal to 0. C = controls, LPS = LPS-stimulated treatments. Statistically significant differences (*p* ≪ 0.001; electronic supplementary material, table S11 in SM1) are marked with asterisks. (Online version in colour.)

To verify these transcriptomic patterns indicating functional effects of *CNR2* loss on neuroimmune functioning in parrots, we followed the *CNR1/2*, *IL1B* and *IL6* expression on messenger RNA (mRNA) level across the two taxa using RT-qPCR. In the budgerigar (E1), relative *IL1B* and *IL6* expression increased in the brain following the LPS stimulation (*p* < 0.001 for both markers; electronic supplementary material, S1, tables S11–S13). By contrast, the expression of *CNR1* was independent of the LPS treatment (*p* > 0.05; electronic supplementary material, S1, tables S11 and S14). Considering the putative interspecific differences, we next compared changes in *IL1B* and *CNR1* gene expression on the mRNA levels following LPS stimulation in the six parrot species (E2). The results confirmed that the expression of *IL1B* in the brain changes in response to LPS stimulation, regardless of species (*p* = 0.005; electronic supplementary material, S1, tables S11 and S15). Again, we found no effect of the LPS stimulation on *CNR1* mRNA expression (*p* > 0.050; electronic supplementary material, S1, tables S11 and S16). By contrast, in the zebra finch, a species with a functional CNR2 receptor, there was no significant effect of the LPS treatment on expression changes of any of these genes (*p* > 0.050; electronic supplementary material, S1, tables S11, S17–S20). There was no correlation between *CNR1* and *IL1B* (*p* > 0.050) or *IL6* (*p* > 0.050) expression in brain in any of the compared taxa (electronic supplementary material, S1, figures S3–S7). However, expression of *CNR2* in zebra finch was significantly positively correlated with expression of *IL1B* (*p* = 0.009; *r* = 0.711; electronic supplementary material, S1, figure S8), but not *IL6* (*p* = 0.753, *r* = 0.315, electronic supplementary material, S1, figure S9). Taken altogether, these results confirm no overall increase in expression of neuroinflammatory markers in the *CNR2*-intact passerines following LPS stimulation, but a contrasting significant upregulation of these markers in the brains of LPS-stimulated *CNR2*-deficient parrots (figure 4*a,b*).

## 4. Discussion

Our search through negative regulators of inflammatory responses revealed that *CNR2* is the only gene consistently missing in parrots being a candidate for their susceptibility to neuroinflammation. We show that the *CNR2* gene has been lost during parrot evolution through chromosomal rearrangements. We found no evidence for compensatory evolution in *CNR1* after *CNR2* loss in parrots and the comparative experimental findings suggest that the gene loss events affect neuroimmune regulation. While in passerines possessing functional *CNR2* (represented by the zebra finch) peripheral inflammation induced by LPS failed to trigger any neuroinflammation (measured on transcriptomic level), in the *CNR2*-deficient parrots we recorded in the brain a significant proinflammatory cytokine upregulation.

LPS-induced activation of the immune system in the periphery can trigger systemic immune responses with neuroinflammatory outcomes [72–74] that in mammals can cause important alterations in behaviour and cognition [75]. This phenomenon has not been recorded in birds, where even high doses of LPS (more than 6 mg kg^−1^ of body mass) trigger only mild and non-lethal inflammation [60,76,77]. However, most immunological data for birds have so far only been generated in poultry (evolutionarily basal Galloanserae lineage), or, to a much lesser extent, in passerine birds. Thus, diversity in avian immune responses to peripheral stimulation remains largely unknown. Of particular relevance is the immune response regulation in species with highly rearranged genomes, such as the parrots [29,31].

Peripheral inflammation can modulate the expression of *CNR*s in both the periphery and the brain, thereby altering neuronal processes and behavioural and cognitive functions [78]. We confirmed *CNR1* expression in the nervous system of birds (both zebra finches and parrots), suggesting its similar regulatory effect on neuronal processes as in mammals. In mammals, leucocyte-modulating *CNR2*, an inhibitor of the proinflammatory cytokine secretion [79], is also expressed in both the brain (microglia) and periphery [45], providing an important anti-neuroinflammatory protection to the brain [18–21]. However, somewhat surprisingly, previous radiographic investigations have revealed no signs of its expression in the brain of budgerigars [80]. Our genome-database search indicated a complete absence of functional *CNR2* genes in all parrot species investigated, which contrasts with its conserved presence in all lineages closely related to parrots (i.e. the falcons, seriemas and passerines, including the zebra finch). We were able to identify putative remnants of the *CNR2* pseudogene in the budgerigar genome, indicating apparent *CNR2* pseudogenization following massive karyotype rearrangements early in parrot phylogeny [29,31]. Interestingly, a comparison of the karyotype localization of passerine *CNR2*-neighbouring genes in the budgerigar and kakapo genomes suggested two presumably independent karyotype rearrangement events in parrots resulting in the *CNR2* loss. The absence of *CNR2* was confirmed through negative results for (i) *CNR2-*targeted amplification attempts in budgerigar gDNA using conserved PCR primers, and (ii) searches through Illumina NextSeq-generated transcriptomes of small intestine in six different parrot species. We consider this as a conclusive support for the complete absence of the *CNR2* gene in parrots, although further research should aim to support this finding on the chromosomal level.

This finding raises the question as to whether a pseudogenization event could have affected the regulation of neuroimmune interactions in parrots. Our positive selection analysis indicates that CNR2 is functionally conserved across the avian taxa. As we found no other *CNR* gene in the parrot genomes aside from *CNR1*, we tested for evolutionary changes in *CNR1* that could be linked to *CNR2* absence. Nevertheless, our selection analysis showed that *CNR1* is also highly conserved throughout vertebrates, with no compensatory selection linked to the *CNR2* loss in parrots. This suggests that *CNR2* pseudogenization could have functional significance. To test this hypothesis, we compared data on systemic inflammation in passerines and parrots, focusing on the putative difference in neuroinflammation-linked cytokine expression caused by the lack of the CNR2 negative regulation in parrots [45,81]. By contrast to the zebra finch, in the *CNR2*-defficient budgerigars, we observed upregulation of expression in proinflammatory cytokines such as *IL1B* and *IL6* in the hyperpallial tissue. The same pattern has been detected across all investigated parrot species, suggesting that parrots in general may be more vulnerable to neuroinflammation than other birds. This is supported by the fact that parrots are exceptionally susceptible to bornavirus-related neuropathy [8,82–84] and also other parrot pathogens including bacteria, viruses and fungi are suspected to frequently cause behavioural disorders [85–87].

Our data, therefore, suggest that *CNR2* loss in parrots could impair regulation which dampens systemic proinflammatory signalling (for example, mediated by IL1B and IL6). Evidence from *CNR2*-knock-out mice showing pronounced immunopathology [88], appears to support our interpretation. Thus, our results promote the hypothesis of regulatory relevance of *CNR2* absence in sensitivity to neuroinflammation and also suggest that parrots could be prone to neurological syndromes.

## 5. Conclusion

In this study, we provide comprehensive evidence for CNR2 absence in parrots and initial results documenting the possible impact of this loss on the regulation of neuroinflammation. Specifically, we observed upregulated proinflammatory cytokine expression in parrot brains, but no similar changes in zebra finches possessing fully functional CNR2. With no apparent compensatory evolution in *CNR1*, parrots lacking functional *CNR2* may be more susceptible to systemic neuroinflammation (e.g. induced by dysbiosis) than other avian species. Our findings do not only provide important insights into variability in susceptibility to immunopathology between species but also offer relevant evolutionary evidence for the functional effects of gene loss events during chromosomal rearrangements. Further research is needed to illuminate possible compensatory mechanisms in parrot immunity, and links to parrot infection ecology and evolution.

## Data Availability

All raw and processed sequencing data generated in this study have been submitted to the NCBI (https://www.ncbi.nlm.nih.gov/) under accession numbers SAMN23963146, SAMN23963147, SAMN23963148, SAMN23963149, SAMN23963150, SAMN23963151, PRJNA751848, PRJNA879979. The data are provided in the electronic supplementary material [89].
